# Supplementary figures and images for: Structural and functional characterization of an otopetrin family proton channel
Source: eLife. 2019 Apr 11;8:e46710. doi: 10.7554/eLife.46710 (PMC6483595; doi:10.7554/eLife.46710)

## Slide 1
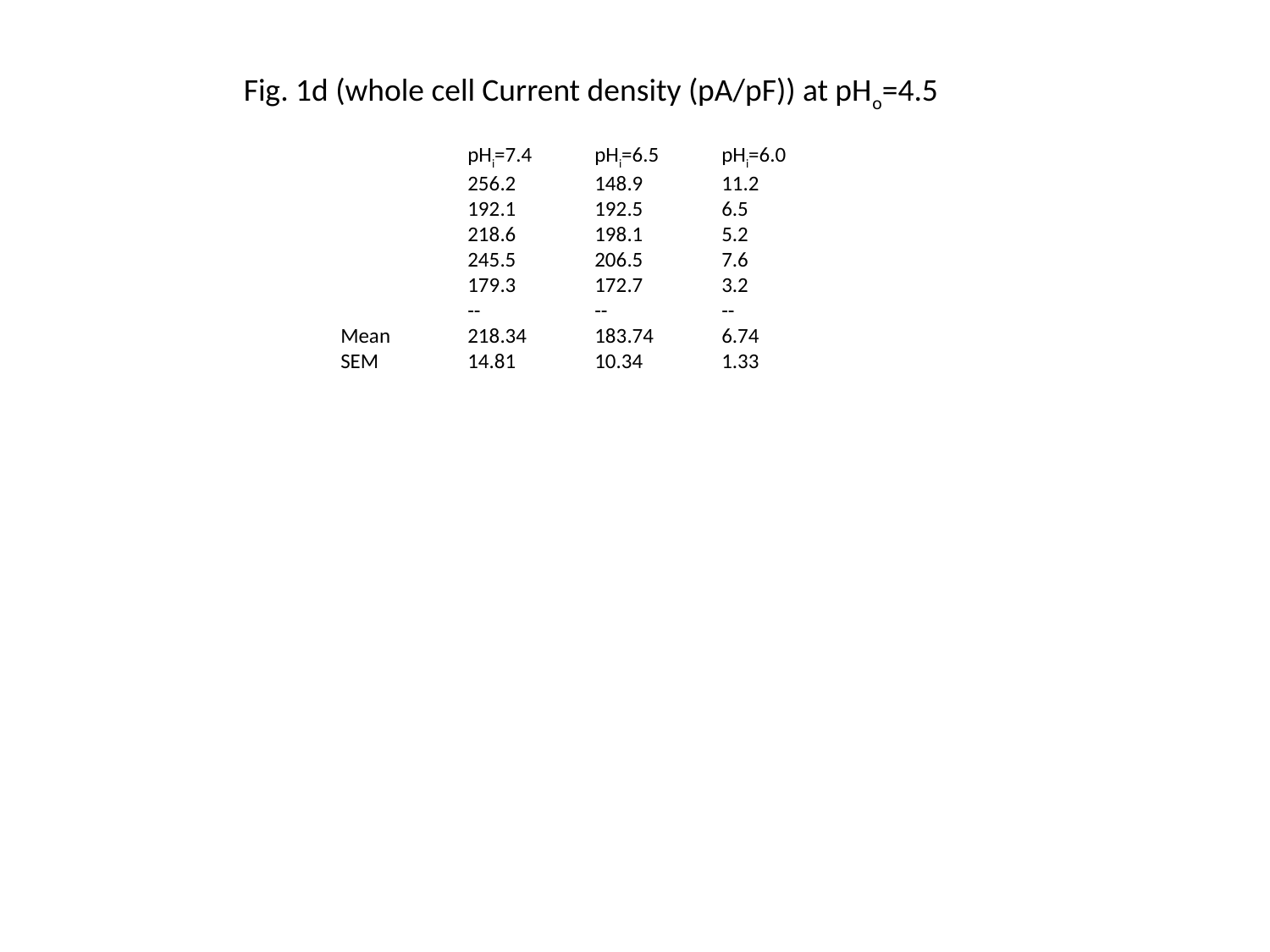

Fig. 1d (whole cell Current density (pA/pF)) at pHo=4.5
	pHi=7.4	pHi=6.5	pHi=6.0
	256.2	148.9	11.2
	192.1	192.5	6.5
	218.6	198.1	5.2
	245.5	206.5	7.6
	179.3	172.7	3.2
	--	--	--
Mean	218.34	183.74	6.74
SEM	14.81	10.34	1.33

Supplement: Figure 1—source data 1. [file elife-46710-fig1-data1.pptx]

## Slide 1
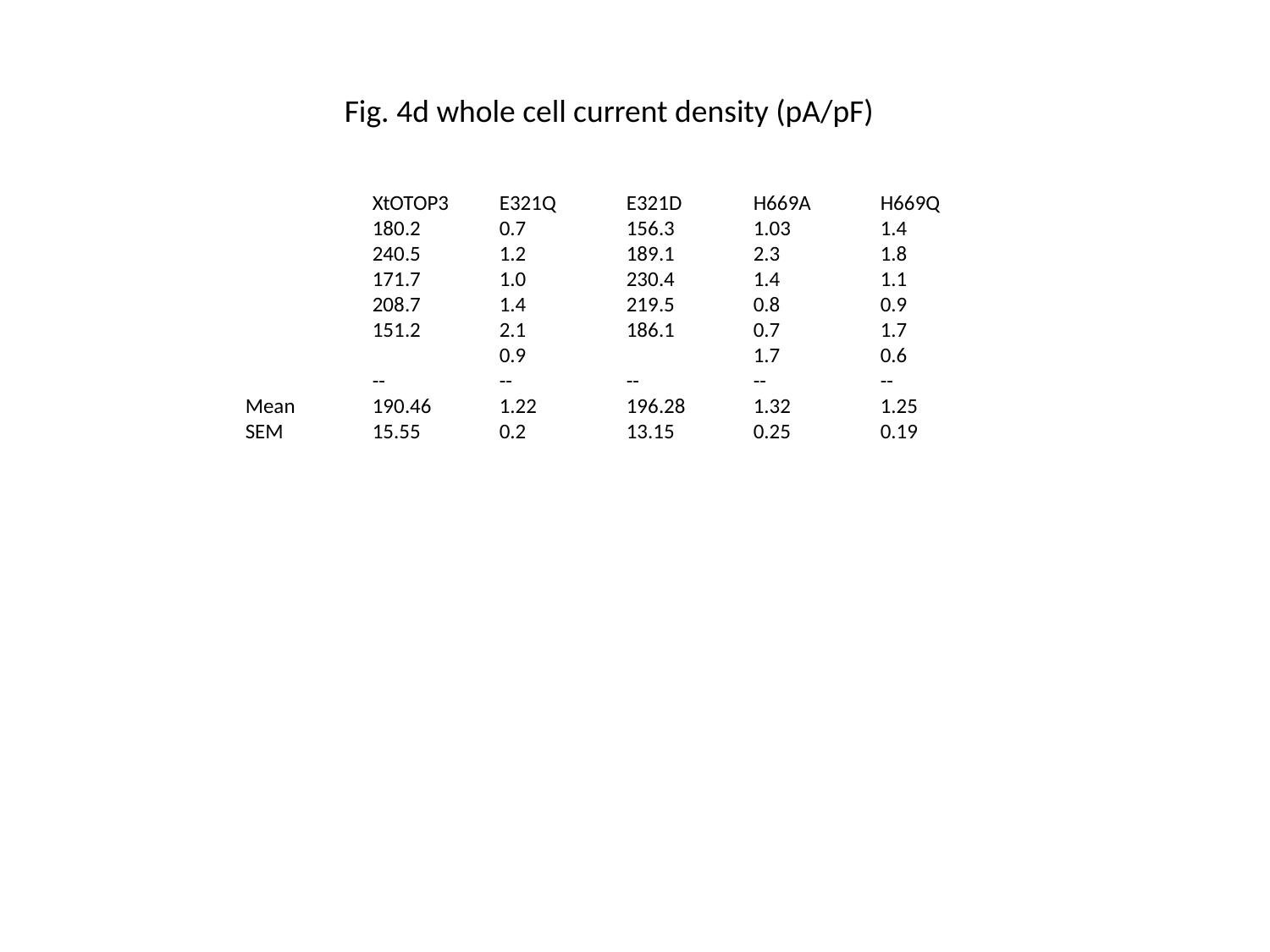

Fig. 4d whole cell current density (pA/pF)
	XtOTOP3 	E321Q	E321D	H669A	H669Q
	180.2	0.7	156.3	1.03	1.4
	240.5	1.2	189.1	2.3	1.8
	171.7	1.0	230.4	1.4	1.1
	208.7	1.4	219.5	0.8	0.9
	151.2	2.1	186.1	0.7	1.7
		0.9		1.7	0.6
	--	--	--	--	--
Mean	190.46	1.22	196.28	1.32	1.25
SEM	15.55	0.2	13.15	0.25	0.19

Supplement: Figure 4—source data 1. [file elife-46710-fig4-data1.pptx]
