# Supplementary material for: Structural and functional characterization of an otopetrin family proton channel
Source: eLife. 2019 Apr 11;8:e46710. doi: 10.7554/eLife.46710 (PMC6483595; doi:10.7554/eLife.46710)
Supplement: Figure 2—source data 1. [file elife-46710-fig2-data1.docx]

**Figure 2-source data 1. Cryo-EM data collection, refinement and validation statistics**
