# Supplementary material for: Structural and functional characterization of an otopetrin family proton channel
Source: eLife. 2019 Apr 11;8:e46710. doi: 10.7554/eLife.46710 (PMC6483595; doi:10.7554/eLife.46710)
Supplement: Figure 3—source data 1. [file elife-46710-fig3-data1.pptx]

## Slide 1
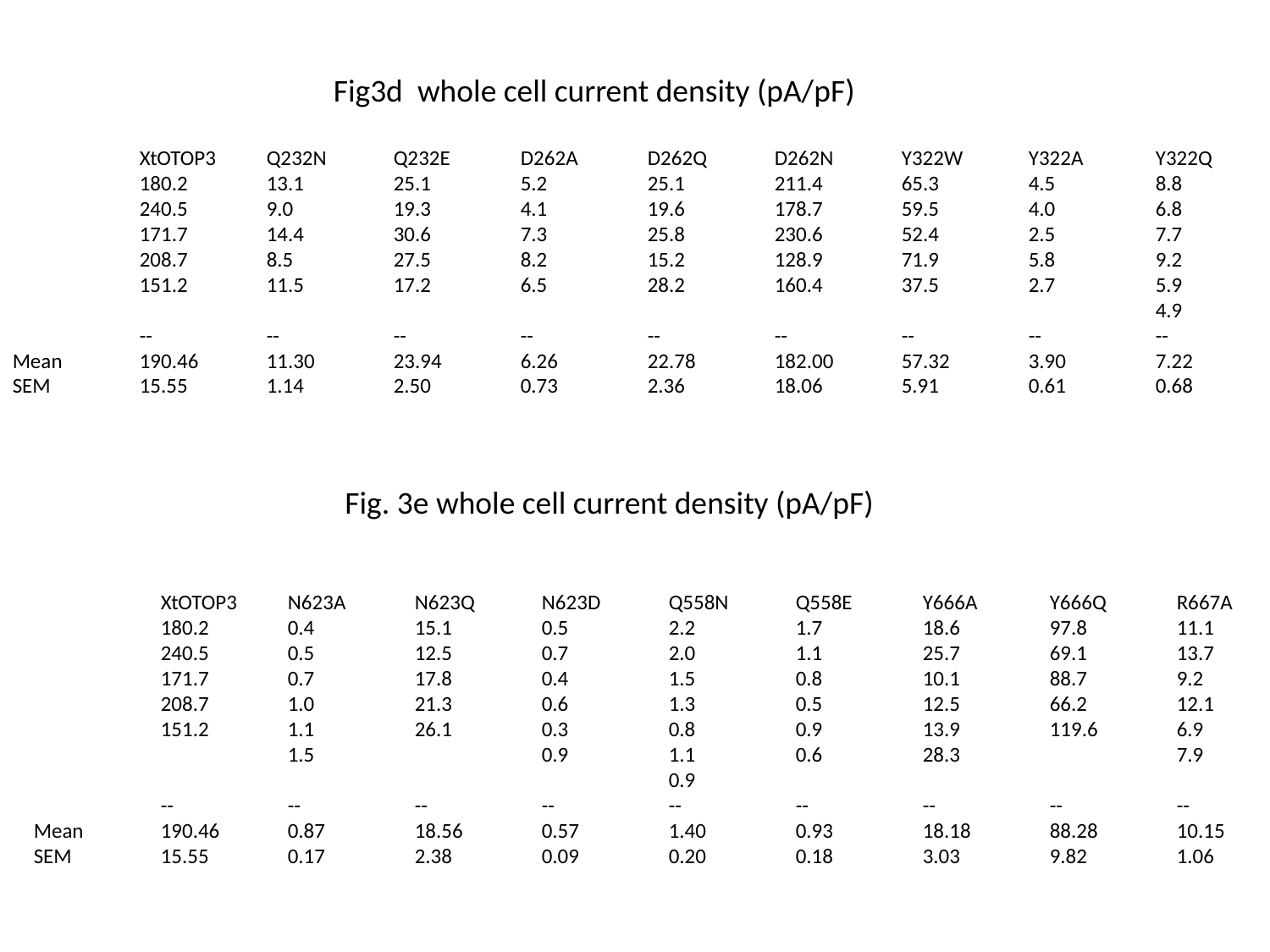

XtOTOP3 	Q232N	Q232E	D262A	D262Q	D262N	Y322W	Y322A	Y322Q
	180.2	13.1	25.1	5.2	25.1	211.4	65.3	4.5	8.8
	240.5	9.0	19.3	4.1	19.6	178.7	59.5	4.0	6.8
	171.7	14.4	30.6	7.3	25.8	230.6	52.4	2.5	7.7
	208.7	8.5	27.5	8.2	15.2	128.9	71.9	5.8	9.2
	151.2	11.5	17.2	6.5	28.2	160.4	37.5	2.7	5.9
									4.9
	--	--	--	--	--	--	--	--	--
Mean	190.46	11.30	23.94	6.26	22.78	182.00	57.32	3.90	7.22
SEM	15.55	1.14	2.50	0.73	2.36	18.06	5.91	0.61	0.68
Fig3d whole cell current density (pA/pF)
Fig. 3e whole cell current density (pA/pF)
	XtOTOP3 	N623A	N623Q	N623D	Q558N	Q558E	Y666A	Y666Q	R667A
	180.2	0.4	15.1	0.5	2.2	1.7	18.6	97.8	11.1
	240.5	0.5	12.5	0.7	2.0	1.1	25.7	69.1	13.7
	171.7	0.7	17.8	0.4	1.5	0.8	10.1	88.7	9.2
	208.7	1.0	21.3	0.6	1.3	0.5	12.5	66.2	12.1
	151.2	1.1	26.1	0.3	0.8	0.9	13.9	119.6	6.9
		1.5		0.9	1.1	0.6	28.3		7.9
					0.9
	--	--	--	--	--	--	--	--	--
Mean	190.46	0.87	18.56	0.57	1.40	0.93	18.18	88.28	10.15
SEM	15.55	0.17	2.38	0.09	0.20	0.18	3.03	9.82	1.06
